# Heavy Metals in Vegetables: Screening Health Risks of Irrigation with Wastewater in Peri-Urban Areas of Bhakkar, Pakistan

**DOI:** 10.3390/toxics11050460

**Published:** 2023-05-16

**Authors:** Mehak Nawaz Khan, Muhammad Anis Aslam, Abdullatif Bin Muhsinah, Jalal Uddin

**Affiliations:** 1 Shanghai Key Laboratory of Hydrogen Science & Center of Hydrogen Science, School of Materials Science and Engineering, Shanghai Jiao Tong University, Shanghai 200240, China; 2 State Key Laboratory of Advanced Special Steel, School of Materials Science and Engineering, International Joint Laboratory of Catalytic Chemistry, College of Sciences, Shanghai University, Shanghai 200444, China; 3 Institute of Chemical Sciences, Gomal University, Dera Ismail Khan 29220, Pakistan; 4 Department of Pharmacognosy, College of Pharmacy, King Khalid University, Abha 61441, Saudi Arabia; 5 Department of Pharmaceutical Chemistry, College of Pharmacy, King Khalid University, Abha 61421, Saudi Arabia

**Keywords:** daily intake, emerging risks, vegetables, heavy metals, Bhakkar

## Abstract

One of the key concerns in public health is food security in the food sector. Due to the large amounts of potentially hazardous metals in wastewater, this practice may pose serious environmental and health risks to neighboring residents. In this study, the health effects of heavy metals in vegetables irrigated with wastewater were studied. The findings indicated a massive accumulation of heavy metals in wastewater-irrigated soil and vegetables collected from Bhakkar, Pakistan. The current study looked at the effects of wastewater irrigation on metal buildup in the soil–plant continuum and the health hazards that come with it (Cd, Co, Ni, Mn, Pb, and Fe). Heavy metal concentrations in vegetables cultivated on soil irrigated with untreated wastewater were not significantly lower (*p* ≥ 0.05) than in vegetables grown on wastewater-irrigated soil and were below the World Health Organization’s recommended limits. A considerable amount of the selected hazardous metals was also swallowed by adults and children who consumed these vegetables, according to the research. On soil that had received wastewater irrigation, Ni and Mn were substantially different at *p* ≥ 0.001 levels. Pb, Ni, and Cd had health risk scores higher than the ones in all ingested vegetables, while Mn had a health risk score greater than the ones in turnips, carrots, and lettuce. The results also showed that both adults and children who consumed these vegetables absorbed a significant amount of the chosen toxic metals. Pb and Cd were shown to be the most dangerous chemical compounds to human health, and everyday consumption of agricultural plants irrigated with wastewater may pose a health risk, according to the health risk criteria.

## 1. Introduction

More than 200 illnesses, from cancer to diarrhea, are brought on by contaminated food that contains dangerous bacteria, viruses, parasites, or chemicals. Because of the estimated 600 million people who get unwell from consuming contaminated food and the 420,000 fatalities globally, 33 million disability-adjusted life years are lost each year. (DALYs). Children under the age of five bear 40% of the weight of food-borne illnesses, which results in 125,000 yearly fatalities. Diarrheal diseases, which yearly infect 550 million people and cause 230,000 deaths, are the most common illnesses caused by consuming contaminated food [1]. Macro and micronutrients are responsible for providing materials that are required for growth. Some heavy metals are classified as xenobiotics since they play no role in body nutrition and can potentially be detrimental in small doses. The presence of these metals is harmful to animals and plants, and their water solubility can create serious environmental problems [2]. About 25.61 percent of the population in Pakistan has access to clean drinking water. Almost a third of Pakistan’s total water resources come from groundwater, which is also the only supply of water in big cities (urban areas make up 30% of the country’s population, while rural areas make up 23.5%). Textile, metal, dyeing chemical, fertilizer, pesticide, petrochemical, cement, energy and power, sugar processing, construction, steel, leather, mining, food processing, and other industries’ products are all included. In Pakistan, there are several big and substantial contributors to surface and groundwater contamination [3]. In certain locations, pollution has increased drastically in recent years, reaching levels that are dangerous to living organisms. Toxic heavy metals are one of the types of pollution that causes the most harm to biological and environmental systems [4].

It is generally recognized that the buildup of potentially toxic elements (PTEs) in soil causes soil deterioration, which can be a severe issue for human health given how quickly metals can accumulate in the body through food, water consumption, and respiration routes. The relevance of ecological risk assessments and their possible effects on human health is underscored by the fact that they might highlight dreadful threats to both the environment and people. Moreover, they necessitate identifying the precise driving forces that contribute to these hazards. By ingestion, digestion, and skin contact, metal buildup in the upper soil poses a hazard to health, and metal levels and nature are strongly connected to soil toxicity by heavy metals. Damage to human health is often unavoidable, including examples of headaches, sleeplessness, insanity, joint pain, and cancer. They can have a greater impact on the filtration of water as well as on soil organisms, plants, and human health [5].

Intoxication with cadmium can induce lung, renal, and skeletal damage, as well as itai-itai illnesses and cancer [6]. Copper is an integral part of the animal liver and the main supporter of copper’s nutritional presentation. The concentration of lead in water ranges from 0.0001 to 2.8 mgL^−1^. According to the European government, the concentration of copper in soil ranges from 4 to 49.26 mg/kg (6 < pH < 7). Copper concentrations in soil in Pakistan range from 6 to 412 mg/kg, with the Kohistan area having the greatest densities. The concentration of copper in water, vegetables, and soil is increased in Pakistan in different places [7]. Iron is a vital component of many proteins, including enzymes and hemoglobin. Groundwater iron concentrations range from 0.01% to 11.8 mg/L. Currently, the average iron concentration in vegetables ranges from 7.28 to 500 mg/kg. Nickel is abundant in nature, and it may be found in plants, animals, and soil. Nickel absorptions in soil range from 4 to 80 parts per million (ppm). The WHO has established a determined boundary of 0.07 mgL^−1^ in drinking water [8].

Among other things, lead poisoning has an impact on both children’s and adults’ IQ, metabolism, renal failure, coma, and death. According to the WHO guidelines, the absolute limit of lead is 0.01 mg/L [9]. Although a little quantity of hazardous metals binds to protein and is transformed into innocuous molecules, levels that exceed endurance capacity can cause serious difficulties. They establish ionic and covalent bonds with key cell constituents, causing damage to the plasmalemma and irregular cellular activity. They can potentially damage the DNA framework, leading to alterations in the genetic composition and gene pool [10]. Chromium is a trace component that exists in nature in oxidation states three and six. Excessive oxygen causes asthma, DNA damage, and cancer by oxidizing the trivalent form to the hexavalent state [11].

The principal sources of chromium in the environment include electroplating, chrome plating, paint polishing, and smelting processes. Plants polluted with chromium cause chromium poisoning in humans, and animal produce causes ulcers, as well as hepatic and kidney cancer. Some studies divided heavy metals into two groups based on their beneficence and noxiousness activities. Some metals, such as lead, cadmium, and chromium, were toxic to organisms while being present in trace amounts. Nickel and manganese, on the other hand, are important elements for organisms in minute proportions; their inappropriate absorption causes minor symptoms, but their high concentration still makes them hazardous [12]. Due to rising urbanization and industrialization, human metal inputs outweigh natural sources. Heavy metals are found in groundwater, surface water, and soils from a variety of sources, including industrial wastes, air deposition from congested cities, and various home wastes [13].

Heavy metals such as cadmium, chromium, and nickel may be present in soil as a result of parent materials during soil formation [14]. Soil serves as a foundation for all living things on the planet. The most significant aspect is that soil serves as a substrate for plant development, recycling nutrients, and additional resources. Heavy metals (HMs) in contaminated rivers and wastewater are absorbed by the soil, causing negative effects on vegetable development. Roots absorb wastewater and nutrients in a solution as they develop in the soil [15]. Heavy metals bind to soil aquatic and soil elements, which are absorbed by plant roots and stored in vegetables [16]. Some plants can absorb significant quantities of metals from the soil. One of them is leafy vegetables, where research revealed that a high degree of soil pollution constituted a possible threat to vegetables growing nearby [17]. These problems have compelled researchers to evaluate the accumulation of heavy metals in numerous fruits and vegetables, such as mangoes and mushrooms, which are significant agricultural goods found in enormous quantities in local East and Southeast Asia markets [18,19]. High levels of untreated wastewater are used in agricultural areas in the Punjab region of Pakistan’s Bhakkar. Contamination of the land and crops, as well as health issues for the local people, pose the greatest concern. As a result, it is vital to analyze the potential health and environmental consequences of using untreated wastewater for crop irrigation in Pakistan. In Bhakkar District, as in other Pakistani cities, wastewater is routinely used for vegetable irrigation with no prior treatment. As a result, the current study’s objectives were to evaluate (i) HM concentrations in municipal wastewater, (ii) potential HM accumulation in the soil–plant continuum, and (iii) potential health dangers to residents from consuming wastewater-irrigated food crops.

## 2. Materials and Methods

### 2.1. Zone Description

**Figure 1:**
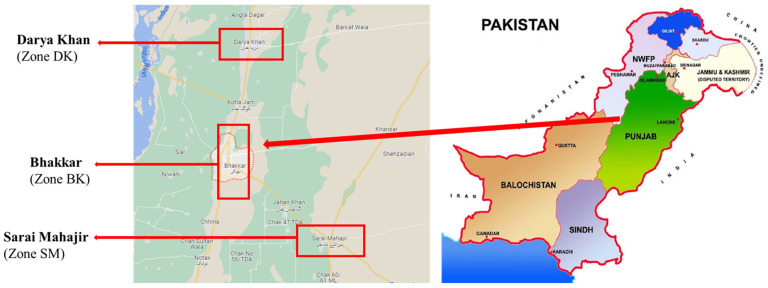
Map of Bhakkar sampling zones.

Bhakkar is a Punjab district well-known for its agricultural products. The present research was carried out in Pakistan’s Punjab Province and Bhakkar District. Bhakkar City is also the administrative center of Bhakkar Tehsil, one of the four tehsils in the district. Within the Bhakkar Tehsil, there are three union councils that make up the city of Bhakkar [20]. Industrial study zones were selected and separated into three sampling zones, i.e., zone-DK (Darya Khan), zone-BK (Bhakkar), and zone-SM (Sarai Mahajir) as shown in Figure 1. The three zones were located nearly 10 km away from each other.

### 2.2. Sample Collection

The sampling locations were divided into three groups. A random sample was taken. Soil samples were collected at a 35 cm depth. Vegetable samples at various stages of development were gathered, and characteristics including pH, TDS, and electrical conductivity (EC) were measured.

### 2.3. Water Sampling

Seventy-two water samples (twenty-four from each zone) were collected at once for the determination of heavy metal concentration and calculation of physical parameters. High-density polyethylene bottles and all glassware were cleansed in 3% nitric acid. De-ionized water was used to wash the objects first. Wastewater samples were collected in 250 mL plastic bottles from various sites and analyzed in a laboratory. Twenty-five milliliters of each sample were moved to a sterile 50 mL container. In a beaker, 5 mL of nitric acid was calculated. It was then processed at 90 °C until it became visible. Whatman no 42-filter paper was used to strain the digestion contents into a measuring flask. Filtrate was created using deionized water and was kept at 5 °C. The pH (pH-7110), EC (HANNA edge EC), and TDS were measured. Heavy metal concentrations (Co, Fe, Mn, Pb, Cd, and Ni) were measured and calibrated using an atomic absorption spectrophotometer. For quality assurance, a non-polluted ionized water sample was utilized as a blank. The analyses of different samples were carried out three times using atomic absorption spectroscopy [21].

### 2.4. Soil Sampling

Three sub-zones within each zone were selected for soil sampling. At a depth of around 30 cm, plants from soil samples were used. Before being pulverized using a pestle, all soil samples were dried in the open air. The mesh size was raised to 1 mm to separate any unwanted pieces, and the samples were kept in plastic bags at 3 °C awaiting testing. The method also includes the preparation of tri-acid solutions: nitric acid at 5%, perchloric acid at 1%, and sulfuric acid at 1%. They were held in the digestion compartment until the evaporation ended. We sifted the digested samples after cooling them. The samples were then placed in a 50 mL measuring beaker and filled the rest of the way with deionized water. Samples were tested for parameters such as pH, EC, and organic matter (OM). Heavy metal concentrations (Co, Fe, Mn, Pb, Cd, and Ni) were measured and calibrated using an atomic absorption spectrophotometer.

### 2.5. Vegetable Sampling

A total of one hundred and eight vegetable samples from various families were collected in labeled polythene bags from the designated zones where soil samples were gathered. Fresh vegetables such as cauliflower and cabbage were collected at random from several Bhakkar localities. The samples were evaluated in a laboratory. After being cleaned with tap water, they were rinsed with water. The edible parts of the vegetables were cut into small pieces and dehydrated in the oven at room temperature. They were broken into well-defined particles with a porcelain mortar and pestle and kept in an airtight polythene bag. The ratio of solution 5:1:1, a tri-acidic solution, and a heated plate were also used to digest the powder of the vegetable samples. The digested samples were filtered using Whatman 42-filter paper into a 20 mL measuring flask, and the filtrate was warmed up with deionized water before being maintained at 5 °C and monitored with an atomic absorption spectrophotometer.

### 2.6. Analysis of Heavy Metals

A questionnaire was used to gather information on people’s weight, family size, ages, vegetable consumption, and vegetable source to perform a nutritional survey and evaluate the risk of consuming wastewater-irrigated vegetables. A total of 150 healthy persons were chosen at random from the population of Punjab, Pakistan Bhakkar area. Three vegetable products were included in the dietary questionnaire, and each was measured in kilograms per person per day using the one-week recall technique. For several vegetables, data on intake frequency and amount were collected. Heavy metal concentrations were measured using an atomic absorption spectrophotometer.

### 2.7. Quality Control

The chemicals used in this experiment were of the highest quality. The water research facility and analytical laboratory at the Institute of Chemical Sciences at Gomal University in D.I. Khan, Pakistan, provided the deionized water for the solutions. All samples were evaluated in triplicate for quality assurance, and blanks and standards were run after each batch of samples.

## 3. Data Analysis

Data were evaluated by using Statistical package for the social sciences (SPSS) software.

### 3.1. Heavy Metal Transfer Factor

Heavy metal transmission and accumulation from soil to crops is a complicated process. The ratio of heavy metal concentration in flora to heavy metal content in soil was calculated using the formula below:

HMTF = C_veg_/C_soil_

Metals in vegetables are represented by C_veg_, whereas metals in soil are represented by C_soil_ [21,22].

### 3.2. Risk Assessment

The hazard quotient (HQ) is a relationship between the computed dose and the reference dose that is used to quantify the risk of metal contamination in vegetables for human health (R_f_D). If the ratio is <1, the population is safe. However, if the figure surpasses or equals one, the population is in serious danger [23].

The HQ was calculated using the formula below:

HQ = [W_plant_] × [M_plant_]/R_f_D × B

[W_plant_] = dry weight (mg·dL^−1^) of ingested vegetables, dry weight (mg·dL^−1^) of consumed vegetables.

[M_plant_] = RfD = metal (mg·dL^−1^) reference dosage in food, metal concentration (mg·kg^−1^) in vegetables.

B = body mass average (kg).

### 3.3. Daily Dietary Index (DDI)

The following formula was used to compute the daily dietary index:

DDI = X × Y × Z/B

where X = vegetables with heavy metals, Y = vegetables’ dry weight, Z = vegetable consumption daily, and B = the average weight of the users.

### 3.4. Daily Intake of Metals (DIM)

The DIM was calculated using the equation

DIM = C_metal_ × C_factor_ × D_food intake_/B_average weight_

where C_metal_ = metal concentration in vegetables (mg·kg^−1^), C_factor_ = conversion coefficient (0.085 for fresh vegetable weight to dry weight), D_food intake_ = daily consumption of vegetables, and B = BMI (body mass index) [24].

### 3.5. Health Risk Index (HRI)

The HRI was computed using the DIM and a reference oral dose as follows:

Health Risk Index = DIM = R_f_D:

If the HRI value is <1, the population exposed is considered safe [25].

## 4. Results and Discussion

### 4.1. Physicochemical Parameters and Concentration Level of Heavy Metals in Wastewater

The physicochemical parameters of the wastewater samples collected randomly from the three zones are shown in Table 1. pH levels in the three zones (DK, BK, and SM) of wastewater varied from 3.20 to 5.09, 3.21 to 4.80, and 3.00 to 5.00, respectively. The WHO’s acceptable pH limit for water is 6.5–8.5. Because waste-tainted water contains more CaCO_3_, NaCl, and Na_2_SO_4_ than wastewater, the pH of wastewater was slightly higher than that of fresh water. The EC of wastewater ranged from 72.0 to 90.0 µS/cm, 65.0 to 87.0 µSc/m, and 75.0 to 97.0 µS/cm, respectively. The EC of wastewater was below the WHO’s limits (1400 µS/cm total dissolved solids (TDS) in wastewater ranged from 43.0 to 97.0 (mg/L), 25.3 to 39 (mg/L) and 35.0 to 80.0 (mg/L), respectively). The TDS of wastewater-contaminated water were below the WHO’s limit of 1000 mg/L. Therefore, fresh water’s EC and TDS are acceptable for irrigation while waste-polluted water is not acceptable for irrigation. A study conducted in Kangal, Andhra Pradesh, found that the pH and EC values in the water and soil were identical [26]. In waste-contaminated water, heavy metals Cd, Co, Fe, Mn, Ni, and Pb had ranges of 0.47–0.87, 0.40–0.60, 0.61–0.95, 0.38–0.87, 0.03–0.85, 0.14–0.19, 7.98–24.67, 8.76–19.23, 8.09–23.60, 0.50–0.62, 0.20–0.52, 6.78–9.26, 5.78–8.45, 2.37–4.14, 1.08–2.31, 9.98–20.50, 0.20–0.50, and 6.78–9.26 (mg/L), respectively, as shown in Table 1. In wastewater, all heavy metals except Co were over the WHO’s permitted levels. Heavy metals in wastewater from the three zones were in the following order: DK zone, Pb > Cd > Ni > Mn > Fe > Co; BK zone, Ni > Cd > Pb > Mn > Fe > Co; and in the SM zone, the order of Cd > Pb > Ni > Mn > Fe > Co differed from that of the fresh water order Fe > Mn > Co > Ni > Cd > Pb.

ANOVA analysis showed that Ni, Cd, Pb, Mn, Fe, and Co concentrations in the present study were not significantly different at *p* ≥ 0.05 levels in wastewater, as shown in Table 2. In waste-contaminated water, the concentrations of heavy metals Cr, Cd, Ni, and Mn ranged from 3.8 to 7.32, 1.80 to 18.20, 0.27 to 0.64, 0.21 to 1.29, and 0.64 to 4.88 mg·L^−1^, whereas fresh water values ranged from 1.32 to 4.12, 0.27 to 1.67, 0.14 to 0.44, 0.05 to 0.20, and 0.12 to 0.72 mg·L^−1^, respectively [27]. These heavy metal concentrations were substantially greater than the results of a previous study: Cd (0.09) mg·L^−1^, Ni (0.06) mg·L^−1^, and Pb (0.03) mg·L^−1^ in wastewater from Varanasi, India, were found at lower levels of concentration [28]. The concentrations of heavy metals Cd, Ni, Pb, and Cr were similarly lower than those reported in [29]. The wastewater from Peshawar’s region (Bara River and Warsak Canal) is not suitable for irrigation due to a high concentration of heavy metals [30].

### 4.2. Physicochemical Analysis and Heavy Metal Content in Soil

The physicochemical parameters of wastewater-irrigated soil samples collected randomly from the three zones are as follows: The pH of wastewater-irrigated soil ranged from 4.90 to 5.09, 3.29 to 5.91, and 3.80 to 5.50; the wastewater-irrigated soil pH was within the range as required by the WHO. Because waste-polluted water contains a high amount of calcium, magnesium, and bicarbonates, the pH of wastewater-irrigated soil was slightly higher than that of fresh water. The EC of wastewater-irrigated soil ranged from 172.0 to 285.0 µS/cm, 180.0 to 250.0 µSc/m, and 175.0 to 189.0 µS/cm. The EC of wastewater was below the WHO’s limits (1400 µS/cm)**.** The EC of wastewater-irrigated soil was not acceptable for irrigation [31]. The amounts of organic matter (OM) in wastewater-irrigated soil were 1.00 to 1.20, 0.60 to 1.10, and 0.30 to 0.50, respectively. Khan et al. investigated the organic matter composition of soil in the Dera Ghazi Khan District, and their findings are in line with the present findings [32].

Heavy metals Pb, Cd, Co, Fe, and Ni in soil irrigated with wastewater ranged from 0.51 to 4.99, 0.7 to 0.26, 0.07 to 1.06, 2.13 to 5.96, and 6.54 to 0.32 to 0.70 mg·kg^−1^ in zone-DK, from 0.83 to 14.06, 0.22 to 1.24, 0.42 to 0.75, 0.27 to 8.40, 1.01 to 11.90, and 0.30 to 1.50 mg·kg^−1^ in zone-BK, and from 2.75 to 19.08, 0.17 to 0.48, 0.31 to 2.16, 1.60 to 4.31, 0.16 to 23 51, and 0.06 to 1.60 in zone-SM. The WHO’s acceptable limits for heavy metals in wastewater-irrigated soil were met. The following heavy metals were discovered in wastewater-irrigated soil in the following order: Pb > Cd > Ni > Fe > Mn > Co, Cd > Ni > Fe > Pb > Mn > Co, and Pb > Fe > Ni > Cd > Mn > Co (Table 3).

ANOVA analysis showed that Ni and Mn were significantly different at *p* ≥ 0.001, and Cd, Pb, Co, and Fe were non-significantly different at *p* ≥ 0.05 levels in wastewater-irrigated soil (Table 4). In this research, irrigation with heavy-metal-contaminated wastewater is primarily responsible for soil pollution. In some studies, except for Cd, which exceeded the permissible limit, in contrast to the current research work, the results obtained were in agreement with the present findings, where the minimum and maximum values for Pb, Cr, Cd, Ni, and Mn ranged from 18.33 to 66.78, 31.65 to 61.65, 7.13 to 11.13, 30.05 to 64.30, and 11.50 to 90.00 and were all within the safe limits of the current research work [27]. In another study, with the exception of Pb, which was above the WHO’s limits, the results obtained, in contrast to the current results, showed that the experiential minimum and maximum values for Pb, Cr, Cd, Cu, Zn, and Ni ranged from 34.90 to 51.80, 30.10 to 38.70, 7.30 to 13.50, 40.10 to 57.30, 48.40 to 59.60, and 38.70 to 45.10, respectively [33].

### 4.3. Heavy Metal Content of Vegetables

**Figure 2:**
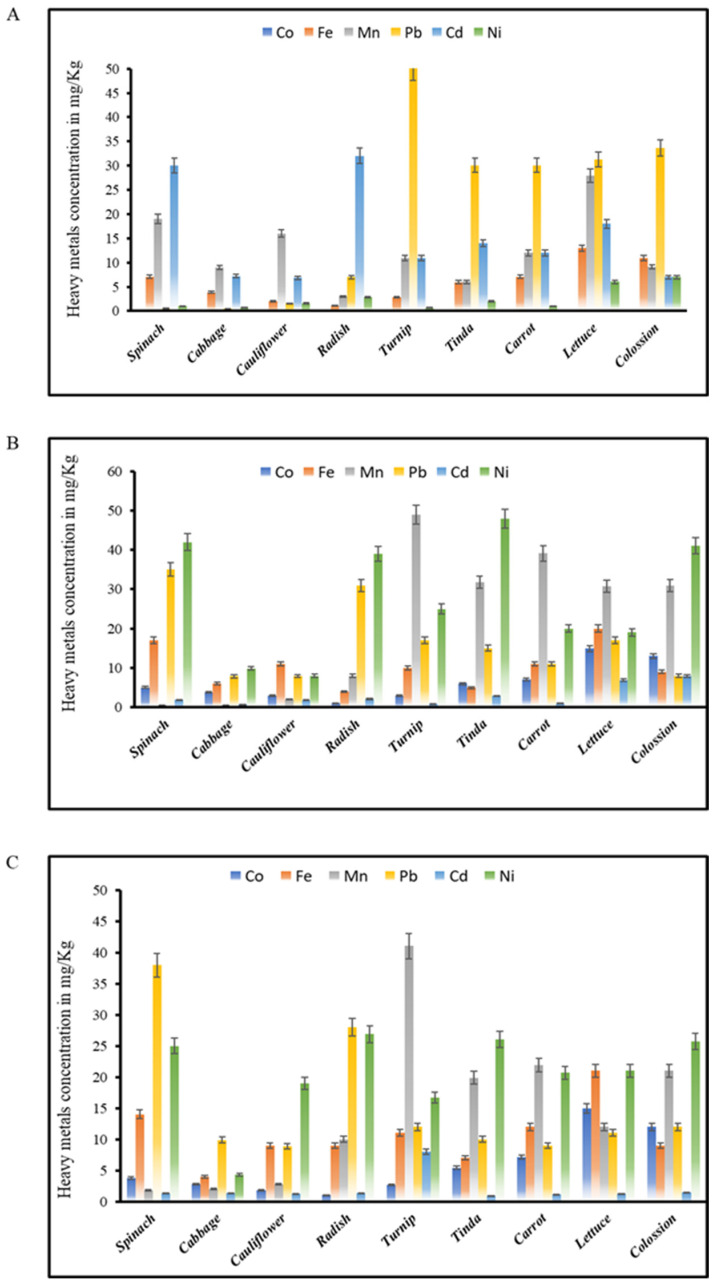
(**A**) Contents of heavy metals in wastewater-irrigated vegetables in zone-DK. (**B**) Contents of heavy metals in wastewater-irrigated vegetables in zone-BK. (**C**) Contents of heavy metals in wastewater-irrigated vegetables in zone-SM.

Heavy metals Co, Fe, Pb, Mn, Cd, and Ni in wastewater-irrigated vegetables from zone-DK ranged from 1.09 to 13.0, 3.0 to 150, 0.42 to 500, 6.89 to 85.0, 0.67 to 7.0, and 6.62 to 67.90 mg·kg^−1^, and those from zone-BK wastewater-irrigated soil ranged from 1.09 to 14.89, 4.0 to 150.0, 0.49 to 500, 7.89 to 85.0, 0.71 to 7.90, and 7.98 to 67 mg·kg^−1^. Heavy metals Co, Pb, Fe, Mn, Cd, and Ni in wastewater-irrigated vegetables from zone-SM ranged from 1.01 to 14.8, 4.0 to 150, 1.78 to 500, 8.90 to 85.0, 0.80 to 8.0, and 4.29 to 67.90 mg·kg^−1^, respectively. Heavy metal elements Pb, Cd, and Ni were found to be above the WHO’s permissible limit when comparing wastewater-irrigated crops to wastewater-irrigated vegetables, having the order of Cd > Pb > Fe > Co > Mn > Ni, Ni > Pb > Mn > Fe > Co, and Pb > Ni> Fe > Mn > Co > Cd, respectively. As shown in Figure 2, Pb, Ni, and Cd concentrations were higher in all nine vegetables *Spinacia oleracea*, *Brassica oleracea var. capitata*, *Brassica oleracea var. botrytis Brassica rapa subsp. Rapa*, *Raphanus sativus*, *Colocasia esculenta*, *Benincasa fistulosa*, *Daucus carota subsp. sativus*, *Lactuca sativa*, and *Daucus carota subsp. sativus*, *Brassica rapa subsp. sativus*, *Brassica rapa subsp. sativus Spinacia oleracea*, and *Benincasa fistulosa*, with Ni having greater concentrations than the other heavy metals. Cd levels were also greater in crops watered with wastewater. In the current research, it was discovered that all crops irrigated with wastewater contained higher levels of the metals under investigation than vegetables irrigated with tube well water. All crops irrigated with wastewater contained heavy metals such as Cd, Pb, and Ni in excess of the WHO’s permitted limit, showing that after the crops were irrigated with wastewater, a buildup in the vegetables took place. Plants cultivated on wastewater-irrigated soils contained heavy metals in excess of the WHO’s allowed levels, suggesting a serious health risk to consumers, according to a prior study [34].

### 4.4. Heavy Metal Transfer Factor

To investigate the human HRI associated with vegetables grown on wastewater-irrigated soil, it is essential to analyze the heavy metal transfer factor. Pb, Cd, Co, Ni, Fe, and Mn concentrations in zone-DK wastewater varied from 0.016 to 0.085, 0.039 to 0.183, 0.058 to 0.532, 0.696 to 3.478, 0.379 to 0.859, 0.294 to 0.656, and 0.247 to 0.924. Water from zone-BK contained varying amounts of Pb, Cr, Cd, Co, Zn, Ni, Fe, and Mn, with values ranging from 0.118 to 0.526, 0.072 to 0.546, 1.000 to 3.810, 0.387 to 0.868, 0.292 to 0.720, 0.250 to 0.954, 0.017 to 0.088, and 0.040 to 0.20. Pb, Cr, Cd, Co, Zn, Ni, Fe, and Mn in wastewater in zone-SM varied from 0.103 to 0.180, 0.117 to 0.807, 0.421 to 0.983, 0.446 to 0.901, 0.185 to 0.874, 0.099 to 0.907, 0.015 to 0.282, and 0.015 to 0.294, respectively, as shown in Table 5. The HMTF trend for heavy metals in all three zones in various vegetables was Cd > Ni > Co > Zn > Cr > Pb > Mn > Fe. Cd, Co, Ni, and Pb had a greater HMTF than the other metals in all wastewater-irrigated crops. The high absorption ratio of heavy metals in wastewater-treated vegetables is attributable to the direct absorption of heavy metals from wastewater, according to the ANOVA study. The current study found higher HMTF values for all metals except Zn and Cr, ranging from 0.04 to 0.11 (Pb), 0.12 to 0.29 (Cr), 0.51 to 1.47 (Cd), 0.32 to 0.51 (Co), 0.36 to 0.57 (Ni), and 0.21 to 0.41 (Zn) mg·kg^−1^, compared to those reported by Khan et al. [35], which could be due to differences in soil with increasing total metal concentrations in soils. Vegetables have also been shown to have an inverse association.

### 4.5. DIM (Daily Intake of Metals) and HRI (Health Risk Index) for Vegetables

The daily dose of heavy metals was calculated based on vegetable consumption. The consumption of agricultural products grown on wastewater-irrigated soil resulted in significantly higher DIM values for heavy metals. Table 6, Table 7 and Table 8 show the DIM from vegetable eating for both adults and children. The DIM in wastewater-irrigated vegetables (zone-DK) of Co, Fe, Mn, Pb, Cd, and Ni ranged from 1.00 × 10^−3^ to 7.00 × 10^−3^, 2.00 × 10^−3^ to 1.50 × 10^−2^, 0.00 × 10^0^ to 2.60 × 10^−2^, 4.00 × 10^−3^ to 1.70 × 10^−2^, 0.00 × 10^0^ to 4.00 × 10^−3^, and 3.00 × 10^−3^ to 2.10 × 10^−2^, respectively, for adults, while for children, it was within the ranges of 1.00 × 10^−3^ to 8.00 × 10^−3^, 2.00 × 10^−3^ to 1.70 × 10^−2^, 0.00 × 10^0^ to 3.00 × 10^−2^, 4.00 × 10^−3^ to 1.90 × 10^−2^, 0.00 × 10^0^ to 4.00 × 10^−3^, and 4.00 × 10^−3^ to 2.40 × 10^−2^, respectively (Table 6). The DIM of Co, Fe, Mn, Pb, Cd, and Ni in zone-BK ranged from 7.00 × 10^−3^ to 1.00 × 10^−3^, 8.00 × 10^−3^ to 2.00 × 10^−3^, 2.60 × 10^−2^ to 0.00 × 10^0^, 1.40 × 10^−2^ to 4.00 × 10^−3^, 4.00 × 10^−3^ to 0.00 × 10^0^, and 2.10 × 10^−2^ to 4.00 × 10^−3^, respectively, for adults, while for children, it ranged from 1.00 × 10^−3^ to 9.00 × 10^−3^, 2.00 × 10^−3^ to 1.20 × 10^−2^, 0.00 × 10^0^ to 3.00 × 10^−2^, 0.00 × 10^0^ to 5.00 × 10^−3^, and 5.00 × 10^−3^ to 2.90 × 10^−2^, respectively (Table 7). The daily intake of Co, Fe, Mn, Pb, Cd, and Ni in zone-SM ranged from 1.00 × 10^−3^ to 8.00 × 10^−3^, 0.00 × 10^0^ to 1.00 × 10^−2^, 1.00 × 10^−3^ to 2.10 × 10^−2^, 5.00 × 10^−3^ to 2.00 × 10^−2^, 0.00 × 10^0^ to 4.00 × 10^−3^, and 2.00 × 10^−3^ to 1.40 × 10^−2^ for adults, while for children, it ranged from 1.00 × 10^−3^ to 9.00 × 10^−3^, 2.00 × 10^−3^ to 1.30 × 10^−2^, 1.00 × 10^−3^ to 2.50 × 10^−2^, 5.00 × 10^−3^ to 2.30 × 10^−2^, 1.00 × 10^−3^ to 5.00 × 10^−3^, and 3.00 × 10^−3^ to 1.60 × 10^−2^, respectively (Table 8). The DIM for adults and children in wastewater-irrigated vegetables was above the bearable daily intake rates for Pb, Cd, and Ni, while it was below the tolerable daily intake rates for all the metals listed in Table 6, Table 7 and Table 8. There was also no harm from eating popular crops cultivated in wastewater-irrigated regions since the DIM levels were under the permissible limits set by the US-EPA and IRIS. The current study’s findings for lead, cadmium, and nickel, which are the most dangerous to human health, were consistent with previous findings [36].

Table 6, Table 7 and Table 8 show the health risk index associated with vegetable consumption for both adults and children. The HRI values in wastewater-irrigated vegetables (zone-DK) for Co, Fe, Mn, Pb, Cd, and Ni ranged from 1.40 × 10^−2^ to 1.70 × 10^−1^, 2.00 × 10^−3^ to 2.10 × 10^−2^, 7.00 × 10^−3^ to 7.95 × 10^−1^, 9.02 × 10^−1^ to 4.19 × 10^0^, 3.51 × 10^−1^ to 3.67 × 10^0^, and 1.73 × 10^−1^ to 1.06 × 10^0^, respectively, for adults, while for children, they ranged from 4.00 × 10^−3^ to 1.96 × 10^−1^, 2.58 × 10^−3^ to 2.41 × 10^−2^, 7.68 × 10^−3^ to 9.15 × 10^−1^, 1.04 × 10^0^ to 4.82 × 10^0^, 4.04 × 10^−1^ to 4.22 × 10^0^, and 2.00 × 10^−1^ to 1.22 × 10^0^, respectively (Table 6). The HRI in zone-BK vegetables for Co, Fe, Mn, Pb, Cd, and Ni ranged from 1.43 × 10^−2^ to 1.95 × 10^−1^, 2.99 × 10^−3^ to 1.50 × 10^−2^, 7.78 × 10^−3^ to 7.77 × 10^−1^, 1.03 × 10^0^ to 4.58 × 10^0^, 3.72 × 10^−1^ to 4.14 × 10^0^, and 2.09 × 10^−1^ to 1.26 × 10^0^ for adults, and for children, it ranged from 1.60 × 10^−2^ to 2.24 × 10^−1^, 3.00 × 10^−3^ to 1.70 × 10^−2^, 9.00 × 10^−3^ to 8.95 × 10^−1^, 1.19 × 10^0^ to 5.28 × 10^0^, 4.28 × 10^−1^ to 4.76 × 10^0^, and 2.41 × 10^−1^ to 1.45 × 10^0^, respectively (Table 7). The HRI in zone-SM vegetables for Co, Fe, Mn, Pb, Cd, and Ni ranged from 1.32 × 10^−2^ to 1.96 × 10^−1^, 2.99 × 10^−3^ to 1.57 × 10^−2^, 2.82 × 10^−2^ to 6.51 × 10^−1^, 1.17 × 10^0^ to 4.97 × 10^0^, 4.77 × 10^−1^ to 4.19 × 10^0^, and 1.12 × 10^−1^ to 7.04 × 10^−1^ for adults, and for children, it ranged from 1.50 × 10^−2^ to 2.26 × 10^−1^, 3.45 × 10^−3^ to 1.81 × 10^−2^, 3.25 × 10^−2^ to 7.49 × 10^−1^, 1.34 × 10^0^ to 5.73 × 10^0^, 5.49 × 10^−1^ to 4.82 × 10^0^, and 1.29 × 10^−1^ to 8.11 × 10^−1^, respectively (Table 8). HRI values for Pb and Cd were more than 1 in all wastewater-irrigated vegetables. *Spinacia oleracea*, *Benincasa fistulosa*, and *Lactuca sativa* all had Ni levels higher than one, posing major health hazards to both adults and children. The HRI values for all heavy metals in fresh-water-irrigated vegetables were 1, presenting no health risks to adults or children.

In nations such as Pakistan, where irrigated wastewater consumption is unregulated, assessing health risks via the food chain is critical. Human contact with heavy metals through food is one of the most common routes, along with air, water, and soil [37]. Similarly, the results achieved for Pb and Cd had HRI values greater than 1, implying that these metals are dangerous to human health even at extremely low doses [27]. The current study’s findings for Pb, Cd, and Ni, which are the most dangerous to human health, were consistent with previous findings [36].

### 4.6. Heavy Metal Correlation Analysis

In wastewater, the physical parameters EC and TDS showed a strong relation and correlation (r = 1). There was a significant correlation at *p* ≤ 0.05 between Co, Cd, Fe (r = 0.141), Mn (r = 0.425), and Pb (r = 0.249). A high correlation at *p* ≤ 0.05 between Co, Fe, Mn (r = 0.516), Pb (r = 0.631), Cd, and Ni was found in wastewater-irrigated soil in [38].

## 5. Conclusions

The measured wastewater parameters indicated a wide range of fluctuation. Pb, Cd, Co, Ni, Fe, and Mn amounts in wastewater, as well as Pb, Cd, and Ni concentrations in vegetables, were found to be above the WHO’s acceptable limit. In the three tested zones, the transfer factor for four heavy elements, namely Cd, Co, Ni, and Zn, was higher. The observed parameters for wastewater showed a significant range of variation. The EC, TDS, and heavy metals Pb, Cd, Co, Ni, Fe, and Mn in wastewater and Pb, Cd, Co, Fe, Mn, and Ni concentrations in vegetables were below the WHO’s permissible limit. The DIM value for adults and children consuming vegetables from the three zones (DK, BK, SM) for three heavy metals (Pb, Mn, Ni) was above the tolerable daily intake rate. The HRI of Pb and Cd was >1 for all the studied vegetables, and the HRI for Ni was >1 in three vegetables, viz. *S. oleracea*, *B. fistulosa*, and *L. sativa*. The studies provide a detailed insight into the present scenario of vegetable contamination and human health risk estimations. To minimize heavy metal buildup in vegetables and, eventually, lower the chronic health risk to the population that consumes vegetables, it is urgently necessary to rigorously monitor the wastewater irrigation system in the research region.

## Figures and Tables

**Table 1:** Physicochemical parameters and concentration level of heavy metals in wastewater.

	Range	Mini.	Max.	Mean	Std. Deviation	Variance	Skewness	WHO (2007)
Zone-DK (Wastewater)								
pH value (H^+^)	2.07	3.02	5.09	4.103	1.038	1.078	−0.415	6.5–8.5
EC (µS/cm)	18.00	72.00	90.00	82.333	9.292	86.333	−1.185	1400
TDS (mg/L)	54.00	43.00	97.00	70.0	27.0	729.0	0.0	1000
Co (mg/L)	0.49	0.38	0.87	0.683	0.265	0.070	−1.597	1
Fe (mg/L)	16.69	7.98	24.67	13.963	9.293	86.371	1.696	5
Mn (mg/L)	0.12	0.50	0.62	0.573	0.064	0.004	−1.545	0.2
Pb (mg/L)	10.52	9.98	20.50	14.860	5.301	28.101	0.632	0.1
Cd (mg/L)	0.40	0.47	0.87	0.706	0.209	0.044	−1.381	0.01
Ni (mg/L)	2.67	5.78	8.45	7.373	1.408	1.982	−1.429	0.2
Zone-BK (Wastewater)								
pH value (H^+^)	1.59	3.21	4.80	3.973	0.797	0.635	0.355	6.5–8.5
EC (µS/cm)	22.00	65.00	87.00	76.667	11.060	122.333	−0.535	1400
TDS (mg/L)	45.00	35.00	80.00	55.0	22.913	525.000	0.935	1000
Co (mg/L)	0.82	0.03	0.85	0.557	0.457	0.209	−1.699	1
Fe (mg/L)	10.47	8.76	19.23	13.110	5.455	29.755	1.306	5
Mn (mg/L)	0.32	0.20	0.52	0.331	0.168	0.028	1.370	0.2
Pb (mg/L)	0.32	0.20	0.52	0.331	0.168	0.028	1.370	0.1
Cd (mg/L)	0.20	0.40	0.60	0.5200	0.106	0.011	−1.458	0.01
Ni (mg/L)	1.77	2.37	4.14	3.20	0.890	0.792	0.548	0.2
Zone-SM (Wastewater)								
pH value (H^+^)	2.00	3.00	5.00	4.0	1.0	1.0	0.0	6.5–8.5
EC (µS/cm)	22.0	75.0	97.0	84.0	11.533	133.0	1.373	1400
TDS (mg/L)	25.0	35.0	60.0	48.3	12.583	158.3	0.586	1000
Co (mg/L)	0.05	0.14	0.19	0.161	0.025	0.001	1.585	1
Fe (mg/L)	15.51	8.09	23.60	16.5267	7.84436	61.534	−0.758	5
Mn (mg/L)	0.49	0.07	0.56	0.267	0.259	0.067	1.446	0.2
Pb (mg/L)	2.48	6.78	9.26	8.267	1.312	1.720	−1.453	0.1
Cd (mg/L)	0.34	0.61	0.95	0.773	0.170	0.029	0.350	0.01
Ni (mg/L)	1.23	1.08	2.31	1.827	0.656	0.430	−1.515	0.2

**Table 2:** ANOVA study of heavy metals in peri-urban wastewater from three distinct areas in Bhakkar, Pakistan.

Zones	Co	Fe	Mn	Pb	Cd	Ni
Zone-DK	0.350 a	17.967 a	0.354 a	9.813 a	0.743 a	3.317 a
Zone-BK	0.485 a	8.332 a	0.308 a	4.513 a	0.523 a	4.262 a
Zone-SM	0.395 a	16.382 a	0.193 a	11.153 a	0.873 a	0.155 a
Significant	ns	ns	ns	ns	ns	ns

Letter a, shows significant differences, ns, not significant.

**Table 3:** Physicochemical analysis and heavy metal content in soil.

	Range	Mini.	Max.	Mean	Std. Deviation	Variance	Skewness	WHO (2007)
Zone-DK (Wastewater-irrigated soil)								
pH value (H^+^)	0.19	4.90	5.09	5.003	0.096	0.009	0.757	-
EC (µS/cm)	113.0	172.0	285	215.667	60.715	369	1.562	-
OM	0.20	1.00	1.20	1.067	0.115	0.013	1.732	-
Co (mg/kg)	0.99	0.07	1.06	0.530	0.499	0.249	0.619	40
Fe (mg/kg)	8.46	6.54	15.00	12.077	4.797	23.015	−1.724	150
Mn (mg/kg)	0.38	0.32	0.70	0.4933	0.192	0.037	0.757	500
Pb (mg/kg)	4.48	0.51	4.99	2.943	2.265	5.130	0.746	85
Cd (mg/kg)	0.19	0.07	0.26	0.160	0.095	0.009	0.467	0.8
Ni (mg/kg)	3.83	2.13	5.96	4.247	1.947	3.789	0.892	67.9
Zone-BK (Wastewater-irrigated soil)								
pH value (H^+^)	2.62	3.29	5.91	4.70	1.321	1.746	0.665	-
EC (µS/cm)	70.0	180.0	250.0	221.67	36.856	1358.3	−1.415	-
OM	0.60	1.10	1.70	1.433	0.305	0.093	0.935	-
Co (mg/kg)	0.33	0.42	0.75	0.620	0.176	0.031	−1.508	40
Fe (mg/kg)	10.89	1.01	11.90	6.347	5.448	29.683	0.179	150
Mn (mg/kg)	1.20	0.30	1.50	0.783	0.633	0.401	1.433	500
Pb (mg/kg)	13.83	0.83	14.66	8.230	6.966	48.523	−0.614	85
Cd (mg/kg)	1.02	0.22	1.24	0.573	0.578	0.334	1.723	0.8
Ni (mg/kg)	8.13	0.27	8.40	4.453	4.070	16.566	0.261	67.9
Zone-SM (Wastewater-irrigated soil)								
pH value (H^+^)	1.70	3.80	5.50	4.7333	0.862	0.743	−0.837	-
EC (µS/cm)	14.0	175.0	189.0	180.7	7.371	54.33	1.415	-
OM	0.30	0.50	0.80	0.633	0.15275	0.023	0.935	-
Co (mg/kg)	1.85	0.31	2.16	0.990	1.018	1.036	1.664	40
Fe (mg/kg)	23.35	0.16	23.51	10.463	11.9142	141.95	0.981	150
Mn (mg/kg)	1.54	0.06	1.60	0.663	0.822	0.676	1.524	500
Pb (mg/kg)	16.33	2.75	19.08	10.38	8.218	67.537	0.579	85
Cd (mg/kg)	0.31	0.17	0.48	0.30	0.161	0.026	1.263	0.8
Ni (mg/kg)	2.71	1.60	4.31	3.33	1.505	2.265	−1.691	67.9

**Table 4:** ANOVA study of heavy metals in peri-urban soil from three distinct areas in Bhakkar, Pakistan.

Zones	Co	Fe	Mn	Pb	Cd	Ni
Zone-DK	0.750 a	13.173 a	0.2733 b	6.913 a	0.257 a	1.33 b
Zone-BK	0.480 a	11.503 a	0.328 b	0.828 a	0.182 a	4.803 ab
Zone-SM	0.065 a	12.593 a	1.443 a	2.823 a	0.272 a	5.268 a
Significant	ns	ns	*	ns	ns	*

Letters a, b shows significant differences; * significant at 0.05; ns, not significant.

**Table 5:** Heavy metal transfer factor (HMTF).

Vegetables	Site	Co	Fe	Mn	Pb	Cd	Ni
*Spinach*	DK	0.224	0.042	0.002	0.383	0.435	0.607
	BK	0.166	0.032	0.002	0.461	0.905	0.660
	SM	0.213	0.228	0.015	0.975	0.898	0.591
*Cabbage*	DK	0.123	0.020	0.001	0.092	0.339	0.131
	BK	0.124	0.009	0.002	0.104	0.338	0.156
	SM	0.157	0.178	0.017	0.254	0.947	0.101
*Cauliflower*	DK	0.063	0.036	0.005	0.088	0.696	0.10
	BK	0.097	0.020	0.007	0.104	0.857	0.126
	SM	0.100	0.282	0.024	0.228	0.849	0.449
*Radish*	DK	0.034	0.007	0.021	0.408	1.261	0.549
	BK	0.035	0.020	0.027	0.408	1.000	0.613
	SM	0.057	0.168	0.087	0.718	0.933	0.636
*Turnip*	DK	0.092	0.025	0.152	0.141	0.291	0.313
	BK	0.095	0.025	0.164	0.224	0.429	0.393
	SM	0.149	0.039	0.356	0.308	5.614	0.395
*Tinda*	DK	0.189	0.013	0.091	0.179	0.869	0.613
	BK	0.193	0.015	0.106	0.197	1.376	0.754
	SM	0.305	0.029	0.173	0.257	0.639	0.615
*Carrot*	DK	0.224	0.027	0.091	0.153	0.426	0.336
	BK	0.228	0.027	0.131	0.145	0.462	0.315
	SM	0.402	0.015	0.189	0.231	0.807	0.489
*Lettuce*	DK	0.411	0.062	0.095	0.230	2.609	0.606
	BK	0.484	0.048	0.103	0.224	3.286	0.299
	SM	0.845	0.119	0.104	0.282	0.849	0.497
*Colossion*	DK	0.348	0.020	0.102	0.089	3.044	0.483
	BK	0.423	0.020	0.104	0.105	3.762	0.645
	SM	0.677	0.025	0.182	0.308	1.011	0.608

**Table 6:** DIM and HRI for adults and children consuming vegetables grown on wastewater-irrigated soil of zone-DK.

Vegetables		Co	Fe	Mn	Pb	Cd	Ni
*Spinacia oleracea*	Adult						
	DIM	4.00 × 10^−3^	1.00 × 10^−2^	0.00 × 10^0^	1.60 × 10^−2^	1.00 × 10^−3^	2.10 × 10^−2^
	HRI	9.28 × 10^−2^	1.42 × 10^−2^	8.73 × 10^−3^	3.93 × 10^0^	5.24 × 10^−1^	1.05 × 10^0^
	Child						
	DIM	4.00 × 10^−3^	1.10 × 10^−2^	0.00 × 10^0^	1.80 × 10^−2^	1.00 × 10^−3^	2.40 × 10^−2^
	HRI	1.07 × 10^−1^	1.64 × 10^−2^	1.01 × 10^−2^	4.52 × 10^0^	6.03 × 10^−1^	1.21 × 10^0^
*Brassica oleracea*	Adult						
	DIM	2.00 × 10^−3^	5.00 × 10^−3^	0.00 × 10^0^	4.00 × 10^−3^	0.00 × 10^0^	5.00 × 10^−3^
	HRI	5.09 × 10^−2^	6.73 × 10^−3^	6.67 × 10^−3^	9.47 × 10^−1^	4.08 × 10^−1^	2.26 × 10^−1^
	Child						
	DIM	2.00 × 10^−3^	5.00 × 10^−3^	0.00 × 10^0^	4.00 × 10^−3^	0.00 × 10^0^	5.00 × 10^−3^
	HRI	5.90 × 10^−2^	7.75 × 10^−3^	7.68 × 10^−3^	1.09 × 10^0^	4.70 × 10^−1^	2.61 × 10^−1^
*Brassica oleracea var. botrytis*	Adult						
	DIM	1.00 × 10^−3^	8.00 × 10^−3^	1.00 × 10^−3^	4.00 × 10^−3^	1.00 × 10^−3^	3.00 × 10^−3^
	HRI	2.62 × 10^−2^	1.20 × 10^−2^	2.48 × 10^−2^	9.02 × 10^−1^	8.38 × 10^−1^	1.73 × 10^−1^
	Child						
	DIM	1.00 × 10^−3^	1.00 × 10^−2^	1.00 × 10^−3^	4.00 × 10^−3^	1.00 × 10^−3^	4.00 × 10^−3^
	HRI	3.00 × 10^−2^	1.38 × 10^−2^	2.85 × 10^−2^	1.04 × 10^0^	9.65 × 10^−1^	2.00 × 10^−1^
*Raphanus sativus*	Adult						
	DIM	1.00 × 10^−3^	2.00 × 10^−3^	4.00 × 10^−3^	1.70 × 10^−2^	2.00 × 10^−3^	1.90 × 10^−2^
	HRI	1.43 × 10^−2^	2.24 × 10^−3^	1.11 × 10^−1^	4.19 × 10^0^	1.52 × 10^0^	9.50 × 10^−1^
	Child						
	DIM	2.00 × 10^−3^	2.00 × 10^−3^	4.00 × 10^−3^	1.90 × 10^−2^	2.00 × 10^−3^	2.20 × 10^−2^
	HRI	4.40 × 10^−2^	2.59 × 10^−3^	1.28 × 10^−1^	4.82 × 10^0^	1.75 × 10^0^	1.09 × 10^0^
*Brassica Rapa. Subsp*	Adult						
	DIM	2.00 × 10^−3^	6.00 × 10^−3^	2.60 × 10^−2^	6.00 × 10^−3^	0.00 × 10^0^	1.10 × 10^−2^
	HRI	3.80 × 10^−2^	8.23 × 10^−3^	7.95 × 10^−1^	1.44 × 10^0^	3.51 × 10^−1^	5.42 × 10^−1^
	Child						
	DIM	4.00 × 10^−3^	7.00 × 10^−3^	3.00 × 10^−2^	7.00 × 10^−3^	0.00 × 10^0^	1.20 × 10^−2^
	HRI	9.00 × 10^−2^	9.48 × 10^−3^	9.15 × 10^−1^	1.66 × 10^0^	4.04 × 10^−1^	6.24 × 10^−1^
*Benicia fistulosa*	Adult						
	DIM	3.00 × 10^−3^	3.00 × 10^−3^	1.60 × 10^−2^	7.00 × 10^−3^	1.00 × 10^−3^	2.10 × 10^−2^
	HRI	7.83 × 10^−2^	4.49 × 10^−3^	4.77 × 10^−1^	1.83 × 10^0^	1.05 × 10^0^	1.06 × 10^0^
	Child						
	DIM	4.40 × 10^−2^	4.00 × 10^−3^	1.80 × 10^−2^	8.00 × 10^−3^	1.00 × 10^−3^	2.40 × 10^−2^
	HRI	4.00 × 10^−3^	5.17 × 10^−3^	5.50 × 10^−1^	2.11 × 10^0^	1.21 × 10^0^	1.22 × 10^0^
*Daucus carota. Subsp*	Adult						
	DIM	4.00 × 10^−3^	6.00 × 10^−3^	1.60 × 10^−2^	6.00 × 10^−3^	1.00 × 10^−3^	1.20 × 10^−2^
	HRI	9.28 × 10^−2^	8.98 × 10^−3^	4.77 × 10^−1^	1.57 × 10^0^	5.13 × 10^−1^	5.81 × 10^−1^
	Child						
	DIM	4.00 × 10^−3^	7.00 × 10^−3^	1.80 × 10^−2^	7.00 × 10^−3^	1.00 × 10^−3^	1.30 × 10^−2^
	HRI	1.07 × 10^−1^	1.03 × 10^−2^	5.50 × 10^−1^	1.81 × 10^0^	5.91 × 10^−1^	6.69 × 10^−1^
*Lectica sativa*	Adult						
	DIM	7.00 × 10^−3^	1.50 × 10^−2^	1.60 × 10^−2^	9.00 × 10^−3^	3.00 × 10^−3^	2.10 × 10^−2^
	HRI	1.70 × 10^−1^	2.09 × 10^−2^	4.97 × 10^−1^	2.36 × 10^0^	3.14 × 10^0^	1.05 × 10^0^
	Child						
	DIM	8.00 × 10^−3^	1.70 × 10^−2^	1.90 × 10^−2^	1.10 × 10^−2^	4.00 × 10^−3^	2.40 × 10^−2^
	HRI	1.96 × 10^−1^	2.41 × 10^−2^	5.72 × 10^−1^	2.71 × 10^0^	3.62 × 10^0^	1.21 × 10^0^
*Colocasia esculenta*	Adult						
	DIM	6.00 × 10^−3^	5.00 × 10^−3^	1.80 × 10^−2^	4.00 × 10^−3^	4.00 × 10^−3^	1.70 × 10^−2^
	HRI	1.44 × 10^−1^	6.81 × 10^−3^	5.35 × 10^−1^	9.16 × 10^−1^	3.67 × 10^0^	8.35 × 10^−1^
	Child						
	DIM	7.00 × 10^−3^	5.00 × 10^−3^	2.00 × 10^−2^	4.00 × 10^−3^	4.00 × 10^−3^	1.90 × 10^−2^
	HRI	1.66 × 10^−1^	7.84 × 10^−3^	6.16 × 10^−1^	1.06 × 10^0^	4.22 × 10^0^	9.62 × 10^−1^

**Table 7:** DIM and HRI for adults and children consuming vegetables grown on wastewater-irrigated soil of zone-BK.

Vegetables		Co	Fe	Mn	Pb	Cd	Ni
*Spinacia oleracea*	Adult						
	DIM	3.00 × 10^−3^	9.00 × 10^−3^	0.00 × 10^0^	1.80 × 10^−2^	1.00 × 10^−3^	2.20 × 10^−2^
	HRI	6.66 × 10^−2^	1.27 × 10^−2^	9.36 × 10^−3^	4.58 × 10^0^	9.95 × 10^−1^	1.10 × 10^0^
	Child						
	DIM	3.00 × 10^−3^	1.00 × 10^−2^	0.00 × 10^0^	2.10 × 10^−2^	1.00 × 10^−3^	2.50 × 10^−2^
	HRI	7.70 × 10^−2^	1.46 × 10^−2^	1.08 × 10^−2^	5.28 × 10^0^	1.15 × 10^0^	1.27 × 10^0^
*Brassica oleracea*	Adult						
	DIM	2.00 × 10^−3^	3.00 × 10^−3^	0.00 × 10^0^	4.00 × 10^−3^	0.00 × 10^0^	5.00 × 10^−3^
	HRI	4.97 × 10^−2^	4.49 × 10^−3^	7.78 × 10^−3^	1.03 × 10^0^	3.72 × 10^−1^	2.59 × 10^−1^
	Child						
	DIM	2.00 × 10^−3^	4.00 × 10^−3^	0.00 × 10^0^	5.00 × 10^−3^	0.00 × 10^0^	6.00 × 10^−3^
	HRI	5.70 × 10^−2^	5.17 × 10^−3^	8.95 × 10^−3^	1.19 × 10^0^	4.28 × 10^−1^	2.98 × 10^−1^
*Brassica var.botrytis*	Adult						
	DIM	2.00 × 10^−3^	6.00 × 10^−3^	1.00 × 10^−3^	4.00 × 10^−3^	1.00 × 10^−3^	4.00 × 10^−3^
	HRI	3.90 × 10^−2^	8.23 × 10^−3^	3.16 × 10^−2^	1.03 × 10^0^	9.43 × 10^−1^	2.09 × 10^−1^
	Child						
	DIM	2.00 × 10^−3^	7.00 × 10^−3^	1.00 × 10^−3^	5.00 × 10^−3^	1.00 × 10^−3^	5.00 × 10^−3^
	HRI	4.50 × 10^−2^	9.48 × 10^−3^	3.64 × 10^−2^	1.19 × 10^0^	1.09 × 10^0^	2.41 × 10^−1^
*Rapanus sativus*	Adult						
	DIM	1.00 × 10^−3^	2.00 × 10^−3^	4.00 × 10^−3^	1.60 × 10^−2^	1.00 × 10^−3^	2.00 × 10^−2^
	HRI	1.43 × 10^−2^	2.99 × 10^−3^	1.27 × 10^−1^	4.06 × 10^0^	1.10 × 10^0^	1.02 × 10^0^
	Child						
	DIM	1.00 × 10^−3^	2.00 × 10^−3^	5.00 × 10^−3^	1.90 × 10^−2^	1.00 × 10^−3^	2.40 × 10^−2^
	HRI	1.60 × 10^−2^	3.45 × 10^−3^	1.46 × 10^−1^	4.67 × 10^0^	1.27 × 10^0^	1.18 × 10^0^
*Brassica rapa.subsp*	Adult						
	DIM	2.00 × 10^−3^	5.00 × 10^−3^	2.60 × 10^−2^	9.00 × 10^−3^	0.00 × 10^0^	1.30 × 10^−2^
	HRI	3.81 × 10^−2^	7.48 × 10^−3^	7.77 × 10^−1^	2.23 × 10^0^	4.71 × 10^−1^	6.55 × 10^−1^
	Child						
	DIM	2.00 × 10^−3^	6.00 × 10^−3^	3.00 × 10^−2^	1.00 × 10^−2^	1.00 × 10^−3^	1.50 × 10^−2^
	HRI	4.40 × 10^−2^	8.62 × 10^−3^	8.95 × 10^−1^	2.56 × 10^0^	5.43 × 10^−1^	7.54 × 10^−1^
*Benincasa fistulosa*	Adult						
	DIM	3.00 × 10^−3^	3.00 × 10^−3^	1.70 × 10^−2^	8.00 × 10^−3^	2.00 × 10^−3^	2.50 × 10^−2^
	HRI	7.80 × 10^−2^	3.74 × 10^−3^	5.04 × 10^−1^	1.96 × 10^0^	1.51 × 10^0^	1.26 × 10^0^
	Child						
	DIM	4.00 × 10^−3^	3.00 × 10^−3^	1.90 × 10^−2^	9.00 × 10^−3^	2.00 × 10^−3^	2.90 × 10^−2^
	HRI	9.00 × 10^−2^	4.31 × 10^−3^	5.80 × 10^−1^	2.26 × 10^0^	1.74 × 10^0^	1.45 × 10^0^
*Daucus carota.subsp*	Adult						
	DIM	4.00 × 10^−3^	6.00 × 10^−3^	2.00 × 10^−2^	6.00 × 10^−3^	1.00 × 10^−3^	1.00 × 10^−2^
	HRI	9.18 × 10^−2^	8.23 × 10^−3^	6.20 × 10^−1^	1.44 × 10^0^	5.08 × 10^−1^	5.24 × 10^−1^
	Child						
	DIM	4.00 × 10^−3^	7.00 × 10^−3^	2.40 × 10^−2^	7.00 × 10^−3^	1.00 × 10^−3^	1.20 × 10^−2^
	HRI	1.06 × 10^−1^	9.48 × 10^−3^	7.14 × 10^−1^	1.66 × 10^0^	5.85 × 10^−1^	6.03 × 10^−1^
*Lactuca sativa*	Adult						
	DIM	8.00 × 10^−3^	1.00 × 10^−2^	1.60 × 10^−2^	9.00 × 10^−3^	4.00 × 10^−3^	1.00 × 10^−2^
	HRI	1.95 × 10^−1^	1.50 × 10^−2^	4.88 × 10^−1^	2.23 × 10^0^	3.61 × 10^0^	4.97 × 10^−1^
	Child						
	DIM	9.00 × 10^−3^	1.20 × 10^−2^	1.90 × 10^−2^	1.00 × 10^−2^	4.00 × 10^−3^	1.10 × 10^−2^
	HRI	2.24 × 10^−1^	1.72 × 10^−2^	5.62 × 10^−1^	2.56 × 10^0^	4.16 × 10^0^	5.73 × 10^−1^
*Colocasia esculenta*	Adult						
	DIM	7.00 × 10^−3^	5.00 × 10^−3^	1.60 × 10^−2^	4.00 × 10^−3^	4.00 × 10^−3^	2.10 × 10^−2^
	HRI	1.70 × 10^−1^	6.72 × 10^−3^	4.92 × 10^−1^	1.05 × 10^0^	4.14 × 10^0^	1.07 × 10^0^
	Child						
	DIM	8.00 × 10^−3^	5.00 × 10^−3^	1.90 × 10^−2^	5.00 × 10^−3^	5.00 × 10^−3^	2.50 × 10^−2^
	HRI	1.96 × 10^−1^	7.74 × 10^−3^	5.67 × 10^−1^	1.21 × 10^0^	4.76 × 10^0^	1.24 × 10^0^

**Table 8:** DIM and HRI for adults and children consuming vegetables grown on wastewater-irrigated soil of zone-SM.

Vegetables		Co	Fe	Mn	Pb	Cd	Ni
*Spinacia oleracea*	Adult						
	DIM	2.00 × 10^−3^	1.00 × 10^−2^	1.00 × 10^−3^	2.00 × 10^−2^	1.00 × 10^−3^	1.30 × 10^−2^
	HRI	4.95 × 10^−2^	1.05 × 10^−2^	2.82 × 10^−2^	4.97 × 10^0^	6.70 × 10^−1^	6.55 × 10^−1^
	Child						
	DIM	2.00 × 10^−3^	8.00 × 10^−3^	1.00 × 10^−3^	2.30 × 10^−2^	1.00 × 10^−3^	1.50 × 10^−2^
	HRI	5.70 × 10^−2^	1.21 × 10^−2^	3.25 × 10^−2^	5.73 × 10^0^	7.72 × 10^−1^	7.54 × 10^−1^
*Brassica oleracea*	Adult						
	DIM	1.00 × 10^−3^	0.00 × 10^0^	1.00 × 10^−3^	5.00 × 10^−3^	1.00 × 10^−3^	2.00 × 10^−3^
	HRI	3.64 × 10^−2^	2.99 × 10^−3^	3.14 × 10^−2^	1.29 × 10^0^	7.07 × 10^−1^	1.12 × 10^−1^
	Child						
	DIM	2.00 × 10^−3^	2.00 × 10^−3^	1.00 × 10^−3^	6.00 × 10^−3^	1.00 × 10^−3^	3.00 × 10^−3^
	HRI	4.20 × 10^−2^	3.45 × 10^−3^	3.62 × 10^−2^	1.49 × 10^0^	8.14 × 10^−1^	1.29 × 10^−1^
*Brassica var.botrytis*	Adult						
	DIM	1.00 × 10^−3^	0.00 × 10^0^	1.00 × 10^−3^	5.00 × 10^−3^	1.00 × 10^−3^	1.00 × 10^−2^
	HRI	2.33 × 10^−2^	6.73 × 10^−3^	4.41 × 10^−2^	1.17 × 10^0^	6.34 × 10^−1^	4.97 × 10^−1^
	Child						
	DIM	1.00 × 10^−3^	5.00 × 10^−3^	2.00 × 10^−3^	5.00 × 10^−3^	1.00 × 10^−3^	1.10 × 10^−2^
	HRI	2.70 × 10^−2^	7.75 × 10^−3^	5.08 × 10^−2^	1.34 × 10^0^	7.30 × 10^−1^	5.73 × 10^−1^
*Rapanus sativus*	Adult						
	DIM	1.00 × 10^−3^	0.00 × 10^0^	5.00 × 10^−3^	1.50 × 10^−2^	1.00 × 10^−3^	1.40 × 10^−2^
	HRI	1.32 × 10^−2^	6.73 × 10^−3^	1.59 × 10^−1^	3.67 × 10^0^	6.96 × 10^−1^	7.04 × 10^−1^
	Child						
	DIM	1.00 × 10^−3^	5.00 × 10^−3^	6.00 × 10^−3^	1.70 × 10^−2^	1.00 × 10^−3^	1.60 × 10^−2^
	HRI	1.50 × 10^−2^	7.75 × 10^−3^	1.83 × 10^−1^	4.22 × 10^0^	8.02 × 10^−1^	8.11 × 10^−1^
*Brassica rapa.subsp*	Adult						
	DIM	1.00 × 10^−3^	1.00 × 10^−2^	2.10 × 10^−2^	6.00 × 10^−3^	4.00 × 10^−3^	9.00 × 10^−3^
	HRI	3.47 × 10^−2^	8.23 × 10^−3^	6.51 × 10^−1^	1.57 × 10^0^	4.19 × 10^0^	4.38 × 10^−1^
	Child						
	DIM	2.00 × 10^−3^	7.00 × 10^−3^	2.50 × 10^−2^	7.00 × 10^−3^	5.00 × 10^−3^	1.00 × 10^−2^
	HRI	4.00 × 10^−2^	9.48 × 10^−3^	7.49 × 10^−1^	1.81 × 10^0^	4.82 × 10^0^	5.04 × 10^−1^
*Benincasa fistulosa*	Adult						
	DIM	3.00 × 10^−3^	0.00 × 10^0^	1.00 × 10^−2^	5.00 × 10^−3^	0.00 × 10^0^	1.40 × 10^−2^
	HRI	7.08 × 10^−2^	5.24 × 10^−3^	3.16 × 10^−1^	1.31 × 10^0^	4.77 × 10^−1^	6.81 × 10^−1^
	Child						
	DIM	3.00 × 10^−3^	4.00 × 10^−3^	1.20 × 10^−2^	6.00 × 10^−3^	1.00 × 10^−3^	1.60 × 10^−2^
	HRI	8.20 × 10^−2^	6.03 × 10^−3^	3.64 × 10^−1^	1.51 × 10^0^	5.49 × 10^−1^	7.84 × 10^−1^
*Daucus carota.subsp*	Adult						
	DIM	4.00 × 10^−3^	1.00 × 10^−2^	1.10 × 10^−2^	5.00 × 10^−3^	1.00 × 10^−3^	1.10 × 10^−2^
	HRI	9.32 × 10^−2^	8.98 × 10^−3^	3.47 × 10^−1^	1.18 × 10^0^	6.02 × 10^−1^	5.42 × 10^−1^
	Child						
	DIM	4.00 × 10^−3^	7.00 × 10^−3^	1.30 × 10^−2^	5.00 × 10^−3^	1.00 × 10^−3^	1.20 × 10^−2^
	HRI	1.07 × 10^−1^	1.03 × 10^−2^	4.00 × 10^−1^	1.36 × 10^0^	6.94 × 10^−1^	6.24 × 10^−1^
*Lactuca sativa*	Adult						
	DIM	8.00 × 10^−3^	1.00 × 10^−2^	6.00 × 10^−3^	6.00 × 10^−3^	1.00 × 10^−3^	1.10 × 10^−2^
	HRI	1.96 × 10^−1^	1.57 × 10^−2^	1.91 × 10^−1^	1.44 × 10^0^	6.34 × 10^−1^	5.50 × 10^−1^
	Child						
	DIM	9.00 × 10^−3^	1.30 × 10^−2^	7.00 × 10^−3^	7.00 × 10^−3^	1.00 × 10^−3^	1.30 × 10^−2^
	HRI	2.26 × 10^−1^	1.81 × 10^−2^	2.19 × 10^−1^	1.66 × 10^0^	7.30 × 10^−1^	6.33 × 10^−1^
*Colocasia esculenta*	Adult						
	DIM	6.00 × 10^−3^	0.00 × 10^0^	1.10 × 10^−2^	6.00 × 10^−3^	1.00 × 10^−3^	1.30 × 10^−2^
	HRI	1.57 × 10^−1^	6.73 × 10^−3^	3.33 × 10^−1^	1.57 × 10^0^	7.54 × 10^−1^	6.73 × 10^−1^
	Child						
	DIM	7.00 × 10^−3^	5.00 × 10^−3^	1.30 × 10^−2^	7.00 × 10^−3^	1.00 × 10^−3^	1.50 × 10^−2^
	HRI	1.81 × 10^−1^	7.75 × 10^−3^	3.84 × 10^−1^	1.81 × 10^0^	8.68 × 10^−1^	7.75 × 10^−1^

## Data Availability

Not applicable.
